# The dynamic capabilities of county-level government in China during the outbreak of large-scale epidemics: a study based on three cases

**DOI:** 10.3389/fpubh.2025.1562915

**Published:** 2025-04-04

**Authors:** Changwei Wei, Juan Wang, Huangyue Wu, Zuying Xu, Jiahao Zhang

**Affiliations:** ^1^School of Public Policy and Management, China University of Mining and Technology, Xuzhou, China; ^2^School of Economics and Management, Huaibei Normal University, Huaibei, China

**Keywords:** COVID-19, county-level government, dynamic capabilities, large-scale epidemics, crisis

## Abstract

**Background:**

After China entered the stage of normalized epidemic prevention and control, county-level government became the leading force and responsible body for prevention and control. In some counties, the epidemic was initially sporadic but later evolved into large-scale transmission. This situation posed a severe challenge to their dynamic capabilities. The dynamic capacity of county-level government largely determines the effectiveness of dealing with large-scale epidemics.

**Methods:**

This study selected three counties with a large-scale outbreak of COVID-19 in 2022 as samples for multi-case analysis, and used insight ability, integration ability, learning ability and innovation ability as dimensions for comparative analysis. Data and information were collected from the official websites of these three county-level governments using the octopus collector.

**Results:**

The dynamic capabilities of county-level government are related to the results of large-scale epidemic prevention and control. This topic has been less explored in existing research. Compared to Si County and Linshui County, Jiutai District clearly lacks dynamic capabilities in large-scale epidemic prevention and control. The different abilities of county-level government play different roles in epidemic prevention and control.

**Conclusion:**

County-level government are at the weakest stress point in the hierarchical structure of China's power system. They are at the forefront of public crisis management playing an important role, which further increase their pressure. Enhancing the dynamic capabilities of county-level government can greatly help them effectively respond to crises and alleviate their vulnerability.

## Introduction

At the beginning of 2020, COVID-19 broke out in Wuhan, China. It was a serious crisis and a severe test for all levels of government. To effectively combat the pandemic, the Joint Prevention and Control Mechanism of the State Council of China (JPCMoSCC) issued *the Guidelines on Taking Science-based, Targeted, Region-specific, and Tiered Measures for COVID-19 Prevention and Control* on February 17.

Subsequently, the number of newly confirmed cases gradually decreased to single digits. By the end of April 2020, epidemiological containment had been achieved in both Wuhan City and Hubei Province. The last hospitalized COVID-19 patient in Wuhan was discharged. Post-containment, the epidemic pattern transitioned to sporadic occurrences with localized clusters (1). That means epidemic prevention and control shifted from comprehensive planning and deployment to precise implementation of policies at the county-level. In practice, county-level administrations were designated as primary units for epidemic risk classification, employing population demographics, and infection metrics to formulate context-specific containment strategies. This administrative reorientation abruptly concentrated pandemic management pressures on local governments. Inadequate epidemic response at this level not only risked public condemnation but also entailed political accountability. To mitigate resource constraints, these administrations were required to implement adaptive containment measures calibrated to real-time epidemiological developments. Empirical evidence suggests that dynamic policy making constitutes an essential mechanism for alleviating resource allocation challenges in public health emergencies.

In the governance structure, local governments such as counties are the most important units (2). On the one hand, these local governments serve as primary interfaces for citizen-state interactions, directly receiving and processing demands from civil society organizations and individual constituents (3). As key implementers of policies, county-level government provide services to citizens and respond to their needs (4). The part of the pressure on local governments is to meet the demands of the public (5). On the other hand, county-level government have relatively limited resources and weaker ability to absorb additional resources. They have less ability to raise revenue through taxation, especially in rural areas where local government finances face challenges (6). Therefore, county-level government sometimes fail to meet the demands of citizens and interest groups due to the lack of management resources, resulting in conflicts (3). Moreover, county-level government are politically marginalized. With the recentralized, vertical, and hierarchical control of power, local governments are weakened in the process of public policy management. A series of problems such as lack of coordination, overlapping, and conflicting competencies may also occur (7). Especially during crises, county-level government lack the power to respond promptly in emergency situations. They need to consult with the central government on actions or countermeasures, which may delay the timing. Additionally, it is difficult for local governments to access emergency resources through effective coordination (8). For example, many local governments faced the problem of insufficient medical resources, isolation facilities, and vehicles during the COVID-19 epidemic (9).

The COVID-19 pandemic necessitates a dual governance capacity framework at the county level, integrating foundational decision-implementation competencies with real-time adaptive responsiveness to epidemiological developments. This operational context underscores the criticality of analyzing dynamic capabilities within local government crisis management systems. Teece and Pisano defined “dynamics” as the changing characteristics of the environment, while “capabilities” emphasized the key role of strategic management in adapting, integrating, and reconfiguring internal and external organizational skills, resources, and capabilities (10). Subsequently, they further pointed out that dynamic capabilities were the abilities of organizations to utilize existing internal and external specific capabilities to cope with constantly changing environments. They explicitly proposed that dynamic capabilities were composed of three dimensions: integration, construction, and reconstruction ability (11). Eisenhardt and Martin expanded the application areas of dynamic capabilities, believing that dynamic capabilities not only play an important role in dynamic environments, but can also have a significant impact on organizations in static environments (12). Although there are inevitably some differences, most scholars believe that dynamic capabilities are the effective development and implementation of new opportunities. It can be seen that it depicts more about the organization's ability to face new situations and problems.

The dimensions within dynamic capabilities are not isolated from each other. The three abilities of sensing opportunities (insight), seizing opportunities (integrating resources), and reconfiguring resources (innovation) interact with each other. They need to be synergized to respond to environmental change (13). When analyzing how learning mechanisms promote the development of dynamic abilities, Zollo argued that knowledge codification learning could improve the efficiency of resource reorganization. He emphasized that learning capabilities provided the knowledge base for innovative capabilities (14). Helfat systematically explored the microfoundations of the dimensions of dynamic capabilities, including resource reorganization and innovation path dependence. The empirical study showed that environmental insights and technological iterations needed to form a linkage through organizational learning (15). Barreto revealed a framework for the interaction of the dimensions of dynamic capabilities (perception, integration, learning, and innovation). Integration capability was proposed as a key mediating variable linking insight and innovation (16). Schilke proposed a multidimensional model of dynamic capabilities (perception, learning, integration, and coordination). They validated the complementarity of the dimensions in the context of environmental turbulence. Insight ability needed to be combined with rapid integration ability to drive innovation. Conversely, the strengths of a single dimension cannot compensate for the weaknesses of other dimensions (17). Wilden argued that learning ability was positively correlated with innovation ability. They emphasized the importance of integration skills in translating insights into innovation (18).

Although the goals and operational processes of the public sector are significantly different from those of private organizations, they are often driven by environmental pressures and have to undergo reforms. Dynamic capabilities can improve the efficiency and effectiveness of public resources, helping governments better fulfill their commitment to providing services (19). Scholars also hold different opinions on the composition of dynamic capabilities in the public sector. Based on the perspective of knowledge management, Wang and Feng divided dynamic capabilities into absorptive, integrative, learning, and innovative dimensions (20). Fernandes identified five different methods for implementing dynamic capabilities: digital ability, knowledge ability, absorptive ability, strategic ability, and resource ability (21). Wirtz et al. believed that dynamic capabilities were the core challenge that governments faced in achieving digital transformation in the digital age, namely the ability to perceive, seize and transform digital technologies (22).

Of course, there are differences in the dynamic capabilities possessed by different levels of the public sector. For example, dynamic capabilities in public value creation differ between the central governments and local governments. The central government has significant advantages in resource integration and cross-regional coordination. However, the central government's response speed is relatively slow in terms of quickly adapting to specific local needs. Local governments are more flexible and responsive at the local level and are able to adjust service provision quickly (23). Hartley explored differences in dynamic capabilities in innovation management across different levels of the public sector (24). The central government drives innovation by virtue of institutional legitimacy, but bureaucratic procedures can inhibit rapid trial and error. Local governments achieve rapid innovation through informal networks and collaboration with social enterprises, but face sustainability challenges. The innovation capacity of central-level government is more in the form of “policy incubation.” This ability is reflected in the “practice diffusion” of local level governments.

Some studies have also found that there are also differences in the dynamic capacities of central ministries and local organizations in crisis response. The strengths of the central department lie in strategic foresight and cross departmental coordination. The weakness of the central government is its slowness to act. In contrast, local institutions are outstanding in their ability to implement quickly. Their problem is that they do not have a sufficient stock of strategic resources (25). Comfort analyzed the differences in collaboration between federal, state and local governments in the United States during the events of September 11th. The federal government (e.g., FEMA) had the advantage of scale of resources. They were able to mobilize national rescue forces quickly. But there was a delay in the transmission of initial information. Local government failed to timely report on-site intelligence. The local government in New York responded quickly to localization. Fire and police departments acted quickly based on local knowledge. Local government resources were fragmented. Cross-sectoral communication systems were also incompatible. As a whole, the advantage of the central government was resource coordination, while the advantage of local governments was situational action. However, the synergy between the two leads to the problem of “information silos” (26).

In the context of crises, dynamic capabilities can be divided into three dimensions: the ability to perceive crises, the ability to seize new opportunities in crises, and the ability to reconfigure resources to respond to crises (27). When dealing with public crisis, the dynamic capabilities that the government needs mainly include the ability to adapt and learn, the ability to coordinate public services and citizen needs, the ability to manage flexible production systems, and the ability to manage data and digital platforms (28). Some scholars also advocated that the government needed strategic emergency planning, analysis, organizational management, and collaboration abilities to effectively respond to crises (29). Although scholars have not reached a consensus on the dimensions of dynamic capabilities, most of their views include perceptual ability, resource allocation ability, and knowledge learning ability, innovation ability. This means that scholars believe that these four abilities are the most important. Based on existing research and crisis situations, this study defined the dynamic capabilities of the government as a capability package that integrates internal and external resources to adapt to the constantly evolving environment and achieve its own functions. This paper analyzed the dynamic capabilities of county-level government from four aspects: insight ability, integration ability, and learning ability and innovation ability.

At present, many studies have applied dynamic capabilities theory to the field of enterprise strategic management. Some scholars introduced it into the study of the public sector. However, no scholars have yet focused their attention on the dynamic capabilities of county-level government. Based on the foundation laid by the above research, this study will expand and supplement from the following aspects. Firstly, this paper paid attention to analyze the problems of China's county-level government during dealing with COVID-19 from the perspective of dynamic capabilities. Secondly, the multi-case comparative analysis was used to analyze how dynamic capacity affects and increases the vulnerability of county-level government in a crisis. It is hoped that the dynamic capabilities of county-level government and their ability to adapt to the external environment will be enhanced.

## Methods and cases

### Research method

Multi-case comparative analysis is a social science research method. Instead of exploring a single case, this research method selects several cases for in-depth analysis. It systematically compares and analyzes multiple cases so as to reveal the commonalities and differences, as well as the underlying causal relationships or patterns behind these cases. This approach helps the researcher to go beyond the limitations of a single case. This approach can refine theories, test hypotheses, or identify new research questions from a broader perspective.

In this study, the multi-case comparative analysis method was used to investigate the impact of dynamic capabilities on the county's handling of COVID-19. Changchun Jiutai District, Si County, and Linshui County were selected as typical cases. This paper collected data and information on the three county-level government' responses to the COVID-19. Using the four dimensions of dynamic capabilities sorted out above as an analytical framework, this paper compared the dynamic capabilities situation of the three county-level government in responding to COVID-19. Through the comparative study, we summarized the challenges faced by different county-level government. We analyzed in depth the role of dynamic capabilities for county-level government in emergency management based on the results of the study.

### Cases selection

According to *the guidance on accurate prevention and control of COVID-19 from the JPCMoSCC* on February 17, 2020, the cumulative number of cases exceeds 50 was a high-risk area. If the number of confirmed cases in a county exceeds 50 in a short term, it is considered that there has been a large-scale outbreak. Based on this, we selected Jiutai District, Si County, Linshui County as typical cases to analyze the dynamic capabilities of county-level government. These three samples have regional representativeness. Jiutai District in Jilin province is located in the northeast of China. Linshui County in Sichuan province is located in the inland southwest region, and Si County in Anhui province is located in the eastern region of China. Although the geographical locations of the three regions are different, the level of economic development is comparable and the differences are not significant. In 2022, the GDP of Jiutai District, Linshui County and Si County were respectively 24.35 billion yuan, 26.74 billion yuan, and 29.324 billion yuan. Additionally, the outbreak of the epidemic in three regions received widespread attention. According to the Baidu Index, the search index for “Si County Epidemic” reached a maximum of 33,040, while the index for “Linshui County Epidemic” was 31,071. Although the index for “Jiutai Epidemic” was only 3,632, the search index for “Changchun Epidemic” was as high as 94,657. Due to Jiutai being a district under the jurisdiction of Changchun City, along with the outbreak in Changchun, there has also been a large-scale outbreak in Jiutai. Therefore, the public's attention to Jiutai was reflected in their attention to the epidemic in Changchun. We can refer to the search index for “Changchun Epidemic.”

Overall, these three cases firstly meet our criteria for defining large-scale epidemics. Secondly, these cases have attracted widespread attention from society and have a certain level of exposure. Finally, three regions represent different areas. They have similar levels of economic development, making them representative as research subjects. Therefore, this paper chooses Changchun Jiutai District, Si County, and Linshui County as typical cases to study.

After the outbreak of COVID-19 in Wuhan, China, in 2020, in addition to the strictest blockade and traffic control in Hubei Province and Wuhan, social prevention, and control measures were also taken nationwide, such as banning assembly activities and controlling public places. The premise of these measures is that at the beginning of the outbreak in 2020, only Hubei Province had a more severe situation, while other provinces in China did not have frequent or severe outbreaks of epidemics. After entering the stage of normalized prevention and control, China has taken different prevention and control measures for each county. According to the comprehensive study of population and disease incidence rate, the counties are divided into low, medium and high-risk levels. Therefore, the main body of epidemic prevention has become the county-level government. The prevention and control situation has shifted from large-scale prevention and control to precise prevention and control led by county-level government. 2022 is a period of normalized prevention and control, which is fundamentally different from the epidemic prevention situation and measures in 2020.

In 2022, the COVID-19 strain had changed. This new strain was generally not highly pathogenic, but it was highly contagious. In epidemic prevention and control, tracking and detecting mutant strains became more difficult. In this context, the phenomenon of concentrated outbreaks of the epidemic in county-level areas was more prominent, because when the epidemic was found to be more serious in the county, the local government immediately adopted strict prevention and control measures. In this study, Octopus Collector captured data and found that after the mutation of COVID-19 virus strains in 2022, large-scale outbreaks occurred successively in Shaanxi, Jilin, Henan, and Shanghai, which was more common in county areas. The mutation of the virus and changes in the social environment posed challenges to the dynamic capabilities of county-level government, testing their ability to quickly mobilize various resources and effectively control the epidemic. Based on the changes in the social situation mentioned above, we selected three cases that occurred in counties in 2022.

The age structure of the population in the three counties is shown in Table 1. In terms of population, Si County has a population of 763,310 according to the seventh national census. There are 707,537 people in Linshui County and 569,976 people in Jiutai District. In the population demographics, individuals aged 65 and above constitute 14.6% in Si County. Comparatively, the proportion of residents aged 65 and above in Linshui County and Jiutai District are higher, at 18.26% and 16.62% of their respective populations. The population density of the three counties is shown in Table 2. Jiutai District has a population density of 169 individuals per square kilometer, while Si County boasts a higher density with 411 people per square kilometer. Linshui County falls in between, with a population density of 371 people per square kilometer. Not only that, Jiutai District, with the smallest resident population, has the highest administrative cost per unit under the same level of public expenditure. Both lower population density and higher administrative cost per unit provide Jiutai District with good conditions for epidemic prevention and control. And in terms of the number of beds in medical institutions per capita and the number of medical personnel per capita, Si County, Linshui County, and Jiutai District are all at the same level. This indicates that the medical conditions in these three districts are comparable. The data provided reveals that the population size, age composition, and medical conditions of Si County, Jiutai District, and Linshui County are largely comparable.

**Table 1:** Age structure of the population in the three counties (China, 2022).

**County name**	**Under 14 years old**	**15–59 years old**	**Over 60 years old**	**Over 65 years old**
Si County	24.2%	58.1%	17.7%	14.6%
Linshui County	19.39%	57.94%	22.67%	18.26%
Jiutai District	11.34%	64.6%	24.04%	16.62%

**Table 2:** Basic fact sheet for the three counties (China, 2022).

**Basic information**	**Si County**	**Linshui County**	**Jiutai District**
Resident population (n)	763,310	707,537	569,976
Area (km^2^)	1,857	1,907	3,371
Population density (n/km^2^)	411	371	169
Fiscal size (billion yuan)	29.32	26.74	24.35
Public health costs per unit (yuan)	1,513.49	1,581.57	1,417.53
Administrative cost per unit (yuan)	8,928.12	7,882.30	10,588.17
Total number of beds in medical institutions	4,524	3,480	3,171
Number of hospital beds per capita	0.0059	0.0049	0.0055
Number of medical staff	4,584	3,488	3,642
Number of medical personnel per capita	0.0060	0.0049	0.0063

The basic situation of the development of the epidemic in the three samples is as follows.

#### Case 1

**Figure 1:**
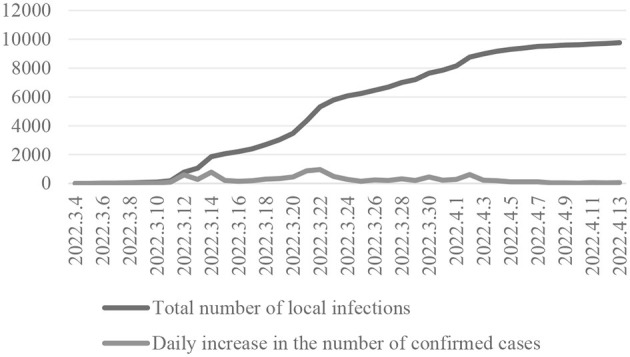
Infection of the COVID-19 in Jiutai District (China, 2022).

At the beginning of 2022, a new round of epidemic broke out in Jilin province, especially in Jiutai District of Changchun City. Since the first positive case was reported on March 3rd, and by April 8th, the total number of reported infections in this region was nearly 10,000 (as shown in Figure 1). On April 27th, Jiutai held a work meeting to gradually lift static control and orderly restore production and living order. As can be seen in Figure 1, the number of new infections per day increased rapidly from March 11 to April 3 in the Jiutai District. This trend continued for about 23 days. After April 3, the increase in the number of new daily infections slowed down.

#### Case 2

**Figure 2:**
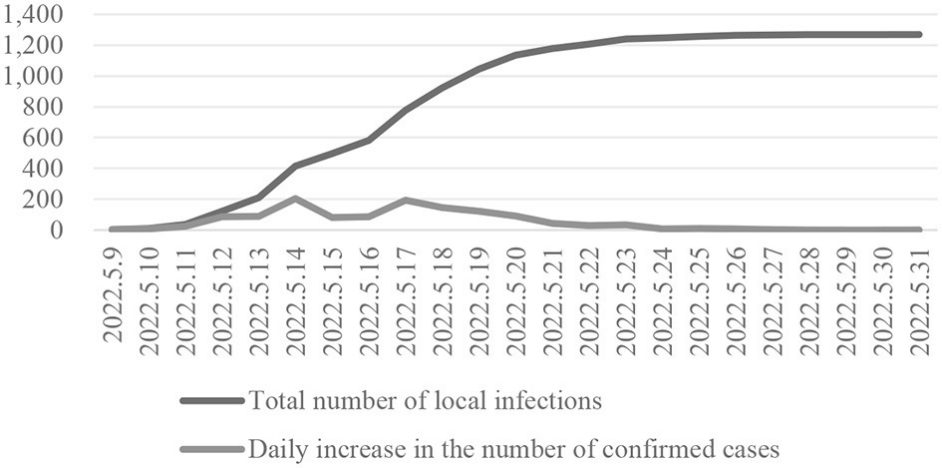
Infection of the COVID-19 in Linshui County (China, 2022).

In May 2022, a sudden COVID-19 caused the small county of Linshui to press the pause button. This epidemic began on May 9th and did not reach zero social coverage until May 22nd. Finally, on May 30th, Linshui gradually lifted static management and orderly restored production and living order. The cumulative number of infections in Linshui exceeded 1,200 cases (as shown in Figure 2). The period from May 11 to May 20 was the most significant period of new daily infections in neighboring counties. This process lasted for 9 days.

#### Case 3

**Figure 3:**
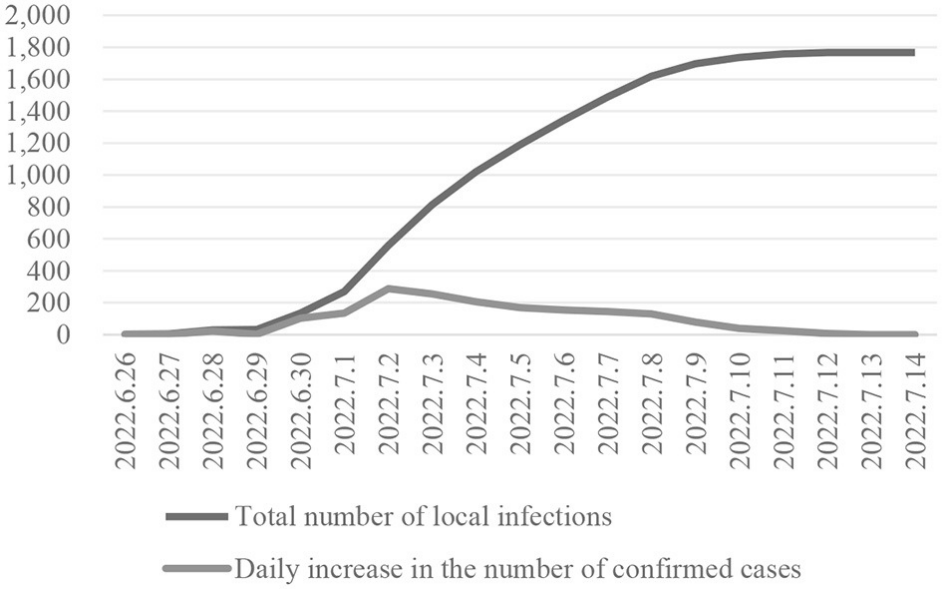
Infection of the COVID-19 in Si County (China, 2022).

In June 2022, a large-scale epidemic broke out in Si County. The first case was discovered on June 26th, and the social coverage was cleared to zero on July 8th. The static management of the entire area was lifted on July 14th. The cumulative scale of infections in Si County this time was 1,767 cases (as shown in Figure 3). Confirmed diagnoses began to appear on June 29 and then continued until July 11, when the number of new daily infections in Si County increased rapidly and remained at a large scale. This period lasted approximately 12 days.

### Data collection

This study used the octopus collector to collect the required data and information. Octopus Collector is a data collection tool based on a graphical interface. It can easily obtain a large amount of standardized data from various websites or web pages in a short period of time. This tool helps any customer who needs to obtain information from web pages to achieve automated data collection, editing, and standardization. Its use reduces the cost of obtaining information and improves efficiency. Octopus Collector is one of the software used by many Chinese scholars for data scraping endeavors (30). This paper used Octopus Collector to capture various information related to epidemic notifications on the official websites of the governments of Si County, Linshui County, and Jiutai District.

**Figure 4:**
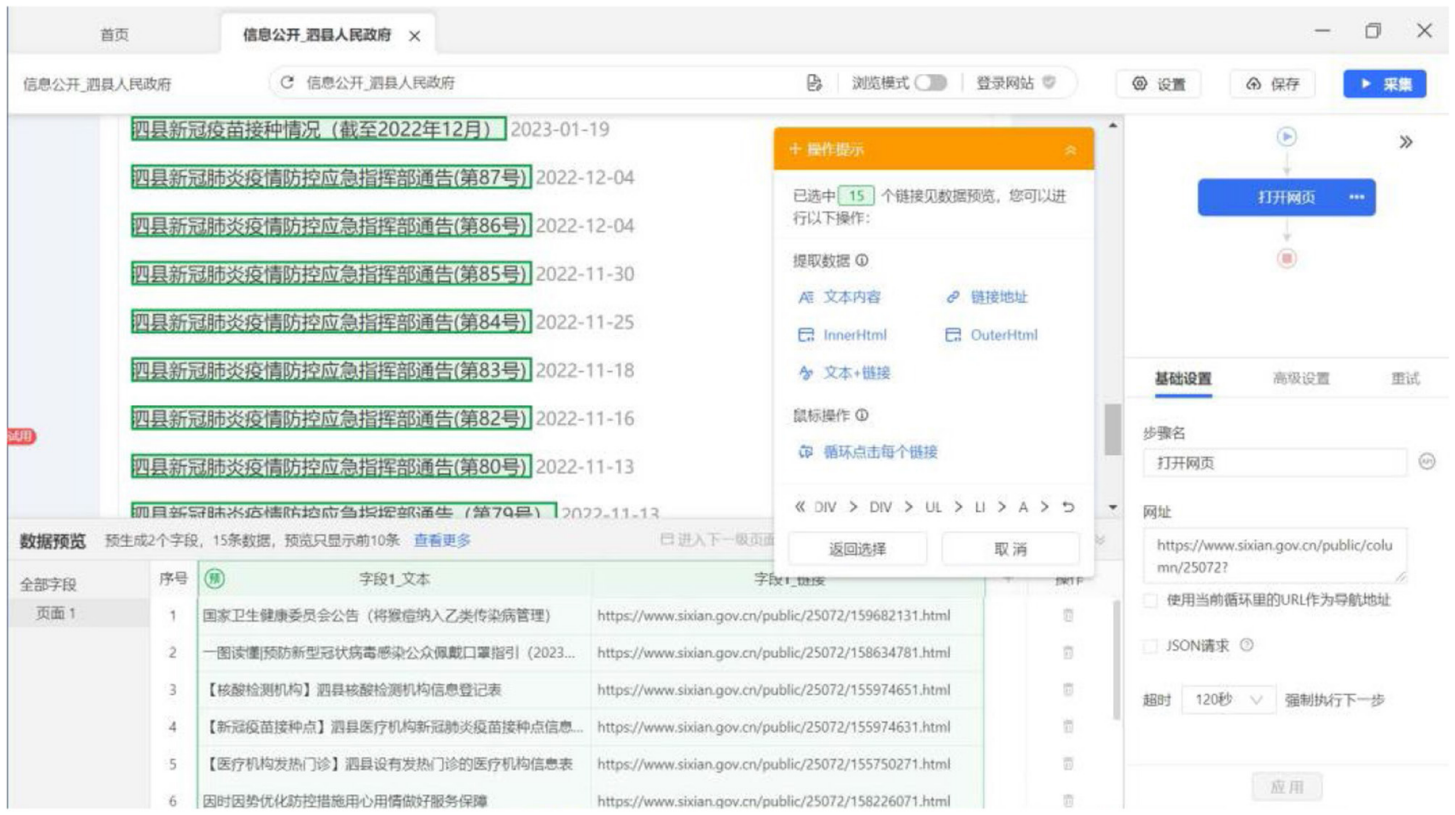
Operation of using octopus collector to collect epidemic data in Si County.

**Figure 5:**
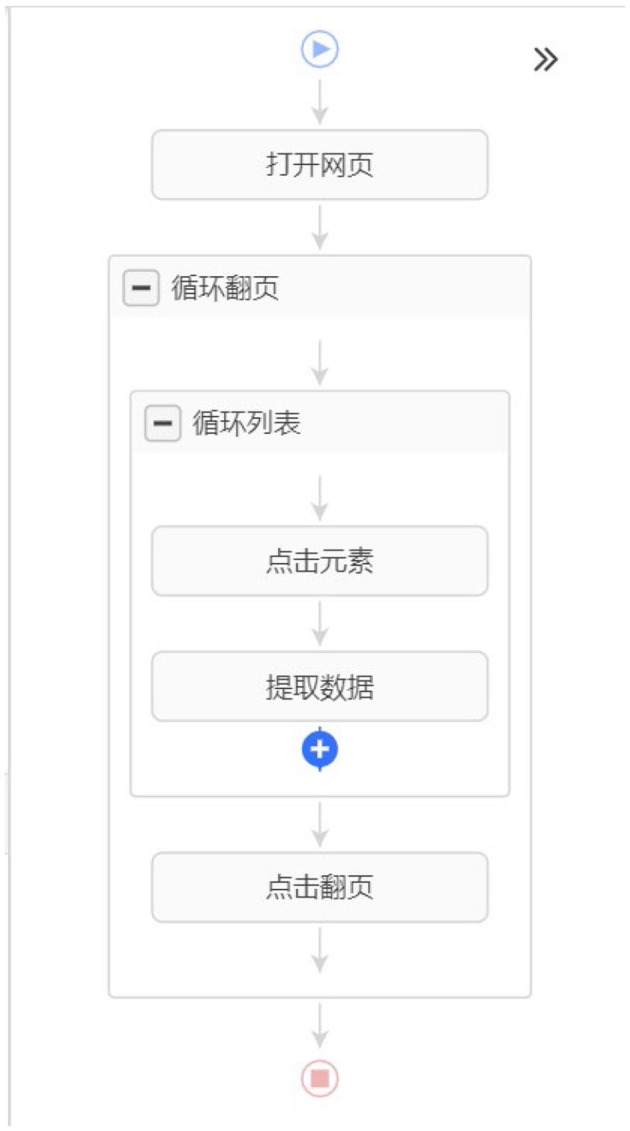
Flowchart of octopus collector collecting data.

This study employs Si County as a case study to elucidate the operational workflow of the Octopus Collector system. First, a new data collection task labeled “COVID-19 Infection Monitoring in Si County” is initialized within the Octopus Collector platform. Subsequently, the target URL corresponding to the Epidemic Prevention and Control announcements published on the official government portal of Si County is entered into the designated URL field. Following initial configuration, systematic rule formulation was conducted through the following technical procedures: Click on the title “Si County New Crown Pneumonia Epidemic Prevention and Control Emergency Response Command Notice” and then click on “Select All Similar Elements.” The Octopus Collector will display the screen in Figure 4. After selecting “Cycle through each element” and coming back, the page will go to the detail page. The operator selects the text that reports the number of new infections and then selects “Text Content.” In order to ensure that the Octopus Collector can automatically turn the page, the operator can click on the “cycle page” in the flowchart, and then click on the “next page.” The automatic page will be set up. The final flowchart is shown in Figure 5. After the rules are set up, the last step is to save the rules. Click the “Start Task” button, Octopus Collector can start to perform the task.

In order to verify the accuracy of the extracted data, this study conducted small-scale test collection before the formal large-scale collection. We selected a small amount of sample data for collection to check whether the collection results were complete and accurate. This verification process mainly included four aspects. The first was to randomly check the integrity of the information to determine whether it contained the expected main fields. The second was to determine whether the obtained information was consistent with the directly visible content on the webpage. The third was to verify the logic of the information, such as whether the information expressed as area, population, fiscal expenditure, and other data conformed to logic. The fourth step was to conduct data comparison, which utilized the data comparison function of Excel to check the differences between the crawled data and the original data. Based on the accuracy results of small sample collection, this paper can initiate large-scale collection. After the collection was completed, this study obtained the number of newly confirmed infections per day in Si County during the epidemic period. We organized the data and made a line graph by summarizing the extracted data through an excel sheet. The epidemic data collection in Jiutai and Linshui County also followed the same steps.

### Indicators and measurement

As mentioned above, four dimensions of dynamic capacities were selected for this study. This paper analyzes the factors that influence county-level government in dealing with COVID-19 in these three areas.

Insight ability means that the governments must be able to grasp the trend and direction of the current state of affairs. When an epidemic occurs, the governments must promptly issue warnings. Timely notices can make the public more alert. *The Emergency Response Law of the People's Republic of China* also stipulates that local governments at or above the county level should promptly issue warnings at the appropriate level (31).

*The Novel Coronavirus Pneumonia Prevention and Control Plan* (ninth version) clearly stipulated that the local joint prevention and control mechanism should release the epidemic situation, risk areas and other relevant information within 5 h after the outbreak of the epidemic. Epidemic information should be based on online direct reporting data. The press conference must be held no later than the following day (32). Therefore, this study uses the duration of warning information and notifications issued by the county government after the outbreak as an indicator. If the county government issued a notice on the day of discovering the epidemic, it can score 25 points in this indicator section. If it issued a notice on the 2^nd^ day, it can score 15 points. If the notice was issued on or after the 3^rd^ day, the score was 0 points. At the same time, the governments should take immediately measures. The ability of the public sector to perceive risk is demonstrated by the speed and responsiveness with which it reacts immediately and delivers services to beneficiaries quickly (33). It was stipulated in the work plan for the prevention and control of COVID-19 during the New Year's Day and the Spring Festival in 2022 that efforts should be made to implement relevant emergency response measures within 24 h (34). Thus, this study selects the time interval between measures taken after the outbreak of the epidemic as an indicator. If the county government took response measures on the day of discovering the epidemic, it can score 25 points. If these measures were taken on the 2^nd^ day, they would receive 15 points. If the interval was 3 days or longer, there would be no score. The ability of governments to think ahead requires that they must act quickly to respond to the uncertainty of an epidemic. As the primary agent of the crisis, the governments must be able to anticipate future scenarios that could jeopardize the system and people's lives (35). The government's insight capacity is not only reflected in the state of crisis, but also in the norm. According to *the Emergency Response Law of the People's Republic of China*, people's government at the county level have the responsibility to carry out systematic investigation, registration and risk assessment of risk sources and hazardous areas, and to implement regular inspection and monitoring (31). Before a crisis occurs, county-level government should take responsibility for identifying sources of danger, and should also conduct regular inventories of emergency supplies. The State Council, in its *National Emergency Response System Plan for the 14*^*th*^ *5-Year Plan*, emphasizes the importance of emergency supplies (36). Based on the above combing of policy texts and related literature, this paper determines whether the government has insight ability based on the following four criteria: (a) The inspection of hazardous sources related to such incidents was carried out prior to the incident. (b) Whether the county-level government regularly conducts an inventory of emergency relief materials. (c) Whether warning information and notification are issued immediately after an incident. (d) The time interval for taking measures after the accident.

Integration ability refers to the ability of the government to access resources at the onset of a crisis, and to be able to integrate internal and external resources at the first opportunity in order to stop the crisis from producing more serious harm. In the process of dealing with COVID-19, the resources that the government has to integrate include financial and human resources. The financial power possessed by the county-level government is firstly reflected in the financial capacity possessed by the county-level government itself. GDP per capita, as a core indicator of a region's economic development level, has been used by some scholars to assess the emergency management capacity of local government (37). When an emergency occurs within a county and exceeds its response capacity, the county-level government may seek necessary assistance and support from the people's governments at the provincial and municipal levels. According to the *Emergency Response Law of the People's Republic of China*, if the county-level people's government is unable to eliminate or effectively control the serious social hazards caused by an emergency, it shall promptly report to the higher-level people's government. The higher-level people's government should take timely measures (31). In the state of emergency, the government's budget allocation for COVID-19 prevention and response is part of the funding for the response to the outbreak (35). Another part of the resources comes from donations of money and goods from the community (33). In turn, the number of health care workers is an important resource for county-level government when responding to COVID-19. Accordingly, this paper chooses the following five indicators: (a) The per capita GDP of the county in the year of the accident. (b) The financial income and expenditure of each county health care commission. (c) The number of social donation amounts received by each county during the epidemic. (d). The number of people served per health technician. (e) Whether assistance is provided by higher levels of government in terms of financial, human and material resources. These five indicators are used to determine whether county-level government have the ability to integrate and coordinate external resources in emergency management.

Learning ability refers to the ability of a government to learn about a crisis to better understand the crisis itself. In order to improve the government's crisis learning ability, the first step is targeted learning and training for government officials. Strengthening the capacity of government officials to deal with public emergencies can improve the overall quality of government officials (38). Secondly, government officials try to learn from past or other national crisis events to deal with unprecedented crisis issues (39). Finally, governments should carry out crisis publicity activities. *The Opinions of the General Office of the Central Committee of the Communist Party of China and the General Office of the State Council on Further Enhancing the Emergency Management Capabilities of Grassroots Levels* mentions that grass-roots governments should carry out extensive public information campaigns (40). The ability to learn is not only in the acquisition of knowledge, but also in the application of knowledge (41). County-level government need to conduct regular emergency exercise activities. Starting from 2021, more and more municipal governments have stipulated that regular drills should be conducted at the county level according to the needs of epidemic prevention and control, with a principle of no less than once per quarter (42). According to this regulation, this study evaluates the frequency of emergency drills conducted by county governments. If the county government had conducted 3 or more drill in the year before the outbreak of the epidemic, the score would be 25 points. If 1–2 drills were conducted, the score would be 15 points. If no drill had been conducted, the score was 0. Therefore, this paper compared the learning ability of county-level government in the following aspects: (a) Number of training courses conducted by county-level government in the year prior to the accident. (b) Number of emergency drills conducted by county-level government in the year prior to the accident. (c) Whether it has taken the initiative to learn from the experience of other incident areas in handling emergencies. (d) Whether to publicize crisis knowledge on the official website.

Innovation ability requires the government not only to systematically organize and deeply integrate existing knowledge. It also needs to reconstruct and innovate its own organizational structure and technical means to better adapt to the continuously changing and developing environment. The innovation ability of the county-level government is firstly reflected in the system innovation. It is the innovation of the emergency plan in the field of emergency management. Due to the scale and severity of the COVID-19 outbreak, government should designate contingency plans based on the changing environment and the needs of beneficiaries. Without contingency plans, organizations will have to reconfigure existing operational capacity and resources in a crisis situation (33). The ability to innovate is also reflected in the innovation of technology. Data empowerment has a significant impact on the effectiveness of government emergency management. The use of big data technology can improve the ability of government to monitor, forecast, early warning, response, collaborate, and communicate (43). The innovative ability of county-level government is also reflected in the innovation of information dissemination channels. *Law of the People's Republic of China on Emergency Response* explicitly proposes to “establish and improve the system of news interviews and reports on emergencies.” This law provides that after the occurrence of emergencies, the relevant people's governments and departments shall promptly publicize to the society the information related to emergencies and the decisions, orders, measures, and other information related to the response to emergencies. The law makes it clear that the government is obliged to release information in a timely and accurate manner through various media channels, including new media, in order to safeguard the public's right to know (31). Organizational innovation is also part of the innovation ability of county-level government. *Law of the People's Republic of China on Emergency Response* stipulates that county-level government may, according to actual needs, set up under this framework relevant categories of emergency response command agencies or working groups and other organizational forms to organize, coordinate and direct emergency response work (31). This study used four indicators to measure whether the government has the ability to innovate: (a) Full use of new media tools for information dissemination and public opinion guidance. (b) Whether the overall emergency response plan was updated within 1 year prior to the accident. (c) Establishment of a special task force across departments within the county-level government after the incident. (d) Establishment of an emergency intelligent management system before the incident.

Cui Li and other scholars assessed the government's emergency management capability in terms of risk perception capability, pre-safety function, monitoring and warning capability, emergency response capability, emotional guidance and reconstruction capability, and emergency audit function. Among them, risk perception ability has the highest weight. Emergency response capability is second (44). Shen also assigned weights to indicators when assessing government emergency preparedness and response capabilities. In his research, command and decision-making capacity was given the highest weight. Rescue capacity was given the second highest weight. Emergency knowledge dissemination as well as emergency training were given lower weights than the first two (45). Based on the above, this study assigned weights to the primary indicators, and the results are shown in Table 3.

**Table 3:** Comparative dimensions of dynamic capabilities.

**Variable name**		**Indicator variables**		**Scoring rules**	
Insight ability	30%	Hazardous Source Identification	Hazardous source inspections related to such incidents were conducted prior to the incident.	Yes	25
				No	0
		Material Inventory	Whether the county-level government regularly conducts an inventory of emergency relief materials.	Yes	25
				No	0
		Early warning notification	Whether warning information and notification are issued immediately after the accident occurs.	Same-day	25
				1-day delay	15
				2-day delay	0
		Measures to be taken	The time interval for taking measures after an accident occurs.	Same-day	25
				1-day delay	15
				2-day delay	0
Integration ability	30%	GDP per capita	GDP per capita for the county in the year of the accident.	Highest	20
				Medium	10
				Least	0
		Epidemic prevention funds	The financial income and expenditure of each county health commission.	Highest	20
				Medium	10
				Least	0
		Social assistance	The number of amounts of social donations received by the county during the outbreak.	Highest	20
				Medium	10
				Least	0
		Number of employees	Number of people served per health technician.	Least	20
				Medium	10
				Highest	0
		Assistance from higher-level government	Whether assistance is provided by higher-level government in terms of financial, human and material resources	Yes	20
				No	0
Learning ability	20%	Crisis communication	Whether to publicize crisis knowledge on the official website.	Yes	25
				No	0
		Emergency Training	Number of training courses conducted by county-level government in the year prior to the accident	3 times and more	25
				1–2 times	15
				No	0
		Emergency Drill	Number of emergency drills conducted by county-level government in the year prior to the accident	3 times and more	25
				1–2 times	15
				No	0
		Specialized Learning	Whether it takes the initiative to learn from the experience of other places and regions in dealing with emergencies.	Yes	25
				No	0
Innovation ability	20%	Institutional Innovation	Whether the overall emergency response plan was updated within 1 year prior to the accident	Yes	25
				No	0
		Organizational Innovation	Establishment of special working groups across departments within the county-level government after the incident.	Yes	25
				No	0
		Technological innovation	Establishment of an intelligent emergency management system before the event.	Yes	25
				No	0
		Information dissemination innovation	Make full use of new media tools for information dissemination and public opinion guidance.	Yes	25
				No	0

## Results

### Insight ability of county-level government

Prior to the large-scale outbreak, the county-level government of all three districts had conducted risk source screening. The outbreak prevention and control command steering group in Jiutai District conducted a field inspection of the outbreak prevention and control work in the urban area on January 19, 2022 (46). Government personnel conducted field inspections of the implementation of standing epidemic prevention and control requirements. Supervisory and inspection activities for epidemic prevention and control were carried in Linshui County on January 30, 2022 (47). Si County also conducted district-wide nucleic acid testing on March 18, 2022. Timely hazard identification enabled county-level government to prepare for emergencies. The governments of all three regions demonstrated a high level of risk preparedness.

Linshui County people's hospital medical supplies reserve warehouse categorized storage of more than 10,000 medical protective masks, 7,000 sets of medical protective clothing, more than 1,800 sets of isolation gowns and other anti-epidemic materials. Si County also requested the Commerce Bureau to map out the county's large shopping malls and supermarkets living supplies reserve situation before the epidemic. This move to ensure the county's supply of essential goods is sufficient. The government of Jiutai District, however, did not conduct an inventory and liquidation of anti-epidemic supplies before the epidemic. This suggests that the government of Jiutai District lacks a dynamic monitoring mechanism for emergency epidemic supplies. Supplies are consumed at different rates in different epidemic prevention scenarios. Without an inventory of the supplies, the rapid consumption of the supplies could not be detected in time. The county-level government's inability to warn and replenish supplies in advance may lead to the risk of supplies being cut off at certain critical points.

There were also slight differences in the time intervals between the release of notification bulletins in the three regions following the outbreak. When the first confirmed case was found on June 26 in Si County, a notice was issued on the same day. In contrast, both Linshui County and Jiutai District released announcements on the government's official website only the day after the first confirmed case was found. Timely release of exact announcements by the government not only raises the alertness of government officials, but also enables the public to take early precautions. Si County's immediate announcement allowed local citizens to be prepared. Citizens reduced the likelihood of contagion by taking steps such as going out less and increasing protection.

The attitudes of these three county-level governments toward taking measures to the first confirmed case varied significantly, with two county-level government promptly implemented measures to prevent and control the epidemic, as shown in Table 4. After the discovery of confirmed case on March 3, Jiutai District did not carry out the first round of nucleic acid testing throughout the district until March 6, and adopted social static management. The local government made an inaccurate assessment of that epidemic, delayed the best control time, and caused the virus to spread for 3 days, resulting in a sharp increase in the number of infections in Jiutai. It was not until 56 days later that the local society returned to normal.

**Table 4:** Insight ability of three county-level government (China, 2022).

**The manifestation of Insight Ability**	**Si County**	**Jiutai District**	**Linshui County**
Start date of the epidemic	2022.6.26	2022.3.3	2022.5.9
Time of publication of the notice	2022.6.26	2022.3.4	2022.5.10
Start date of static management	2022.6.26	2022.3.6	2022.5.10
Interval duration	0 day	3 days	1 day
Date of social zeroing	2022.7.8	2022.4.8	2022.5.22
Date of removing static management	2022.7.14	2022.4.27	2022.5.30
Duration of the epidemic (time from start to removing static management)	19 days	56 days	22 days
Total number of infected individuals	1,767	10,000+	1,269
Number of health technicians	4,584	3,642	3,488
Number of people served per health technician	167	157	203

After the first confirmed case appeared in Linshui County on May 9th, closed static management was implemented throughout the county from the 2^nd^ day onwards, and large-scale nucleic acid testing was carried out in the county's urban area. By comparison, after the first confirmed case was discovered on June 26th, Si County immediately implemented control measures throughout the county, conducting the first nationwide nucleic acid test, implementing differentiated prevention and control, strengthening social control, and strictly limiting gatherings of people. Comparatively, Jiutai District's delayed response—taking control measures only 3 days after the first confirmed case—allowed the virus to spread locally, exacerbating the severity of the outbreak.

Based on the actual performance of the three county governments in various indicators in Table 5, this study evaluates their insight ability. Among them, Linshui County has the highest score. The rating of Jiutai District is the lowest. This indicates that the insight ability of Jiutai District is relatively weak. As shown in Table 5, Jiutai District has a significant gap in taking action compared to the other two counties. This results in a lower overall rating for insight ability.

**Table 5:** Insight ability assessment of three counties.

**Indicators**		**Si County**	**Linshui County**	**Jiutai District**
Insight ability	Hazardous source identification	25	25	25
	Material inventory	0	25	0
	Early warning notification	25	15	15
	Measures to be taken	25	15	0
Aggregate score		75	80	40

### Integration ability of county-level government

The epidemics in Si County, Linshui County, and Jiutai District all occurred in 2022. As can be seen in Table 6, the GDP per capita in Si County in 2022 was 0.039 million. Jiutai District's GDP per capita in 2022 was 0.032 million. Linshui County's GDP per capita in 2022 was 0.038 million. The GDP per capita of Jiutai District in 2022 was the least.

**Table 6:** Health revenues and expenditure and donations of the three county health committees (unit of money: million yuan) (China, 2021–2022).

**Health Revenues, Expenditure and Donations**	**Si County**	**Jiutai District**	**Linshui County**
GDP per capita in 2022	0.039	0.032	0.038
Income in 2022	1,093.91	820.76	1,136.32
Expenditures in 2022	1,155.26	807.96	1,119.02
Income in 2021	1,320.36	785.60	992.08
Expenditures in 2021	1,335.28	777.28	966.51
Donations	17.98	14.21	15.58

Since 2020, Chinese governments at all levels have become familiar with the operation of epidemic prevention, especially with clear expectations for resources and funding investment in epidemic prevention. The epidemic in all three counties occurred in 2022. Table 6 shows that the health expenditure in Linshui County and Jiutai District in 2022 was higher than that in 2021, while the health expenditure in Si County remained stable between 2021 and 2022. Overall, the health expenditure of three counties remained basically unchanged in 2022. In 2021 and 2022, the public health income and expenditure in Jiutai District were the lowest among the three regions, and were significantly lower than those of the other two.

The resources and funding required for epidemic prevention are undoubtedly enormous. In addition to government finances, resources from enterprises and society are also crucial for combating the epidemic. So, governments across China actively mobilize and seek support from external resources. During the large-scale outbreak of the epidemic, these three governments all received epidemic prevention materials and donations from enterprises and society. According to official information, Si County, Linshui County and Jiutai District respectively received social donations of 17.98 million yuan, 15.58 million yuan, and 14.21 million yuan during the epidemic. The donations received by Jiutai are the lowest among the three regions. This means that Jiutai District had a poor ability to absorb external resources. It had a weaker ability to coordinate internal and external resources, and was unable to timely draw on relevant resources to deal with the epidemic. So, it was difficult to purchase sufficient medical equipment and protective materials, and the construction of grassroots medical infrastructure was relatively lagging. These practical problems made Jiutai face greater difficulties in responding to the spread of the epidemic.

In terms of employees, by the end of 2021, there were a total of 4,584 health technicians in Si County, with an average of 167 people equipped with one medical staff. Jiutai District had 3,642 health technicians, with an average of 157 people equipped with one medical staff. Linshui County had the least number of medical staff, with 3,488, and an average of 203 people were equipped with one medical staff, as shown in Table 4. In comparison, there was no significant difference in the number of medical staff between Jiutai District and the other two counties. Due to the relatively small permanent population in Jiutai District, the average number of people served by each medical staff is also lower compared to the other two counties. There is no significant difference in the number of health technicians between Jutai District and the other two counties. Linshui County has the least number of health technicians. Jiutai District has a slightly higher number of health technicians than neighboring counties. Si County has the highest number. Due to the small permanent population in Jiutai District, the average number of people served per health technician is the lowest. This indicates that Jiutai District has the most adequate number of employees among the three districts.

During the response against the large-scale epidemic, Si County, Linshui County, and Jiutai District all received strong support from higher levels of government in terms of funding, manpower and material resources. During the outbreak in Si County, the governments of Anhui Province and Suzhou Municipality quickly deployed financial resources. These funds were used for key aspects such as material procurement, nucleic acid testing and patient care. The provincial government drew medical personnel from across the province to form a medical team to support them. A large number of protective clothing, masks, testing reagents, and other anti-epidemic materials were also delivered in a timely manner to build a solid material foundation for the fight against the epidemic. In Linshui County, the Sichuan Provincial Government quickly responded to increase financial inputs to protect the epidemic prevention and control funding needs. A number of medical teams and streaming teams from the province went to Linshui County to help carry out nucleic acid testing and streaming work. At the same time, provincial and municipal governments actively coordinated. Various kinds of living materials and epidemic prevention materials were constantly transported to neighboring water to meet the needs of residents' lives and the front line of epidemic prevention. During the epidemic in Jiutai District, the Jilin Provincial and Changchun Municipal Governments gave their full support and promptly allocated special funds for epidemic prevention and control expenditures. Medical personnel, community workers, and volunteers were deployed to enrich the anti-epidemic team. They also made every effort to guarantee the supply of epidemic prevention materials and living materials. Such assistance ensured that residents' lives were stabilized and the epidemic containment efforts progressed effectively. The official websites of the three governments did not publish information on the exact amount of support from higher levels of government. However, all three county-level government received assistance from higher government departments.

In the integration ability assessment results shown in Table 7, Si County has the highest score, while Jiutai District has the lowest score. In terms of financial capacity and mobilization of social donations, Jiutai District has the worst performance among the three counties. This result indicates that the integration ability of Jiutai District is relatively weak.

**Table 7:** Integration ability assessment of three counties.

**Indicators**		**Si County**	**Linshui County**	**Jiutai District**
Integration ability	GDP per Capita	20	10	0
	Epidemic Prevention Funds	10	20	0
	Social Assistance	20	10	0
	Number of Employees	10	0	20
	Assistance from higher-level government	20	20	20
Aggregate score		80	60	40

### Learning ability of county-level government

During the period from 2021 to 2022, Si County, Linshui County, and Jiutai District each had different performances in terms of prevention and control emergency drills and training. Si County conducted three emergency drills for COVID-19 prevention and control during 2021–June 2022. Of these, one was organized by the county-level government and one each by Huangwei Town and Changgou Town. In addition, the Si County government organized one training course on the prevention and control of key infectious diseases and the management of epidemic reporting. In Linshui County, seven drills were held from 2021 to May 2022. The government of Linshui County organized 3 county-wide drills, and the remaining drills were conducted by some townships and schools. However, the Linshui County government did not conduct relevant training for government personnel. In contrast, Jiutai District held a single emergency simulation exercise during the 2021–2022 outbreak. The Jiutai District government did not train government personnel on infectious disease prevention and control and outbreak reporting management.

Before the epidemic, Si County held a management training course on the prevention and control of key infectious diseases and epidemic reporting. The training was used to improve the working ability of the management personnel in Si County. Although Linshui County did not explicitly conduct training related to COVID-19, they organized a study of the Animal Disease Prevention and Control Law of the People's Republic of China. In addition to training officials, Linshui County held several meetings of the leadership team for the response to COVID-19. The meetings incorporated best practices from Chengdu and Luzhou municipal responses. Jiutai District Government did not train government officials. This shows that both Si County and Linshui County had a sense of crisis learning. In contrast, the Jiutai District government lacked institutionalized crisis preparedness training.

County-level government in all three regions have been actively launching knowledge campaigns on COVID-19. Linshui County organized community staff to conduct a comprehensive door-to-door survey of family homes in the district. Staff distributed outbreak awareness materials in the district. Jiutai District's official government website had a column on epidemic prevention and control guidelines, which displayed several defense guidelines for COVID-19. A lot of knowledge about epidemic prevention and control was also posted on the WeChat official account of Si County. This shows that the governments of the three regions not only focus on the learning of the government itself, but also pay attention to the public's learning about the crisis.

Furthermore, information on epidemic prevention practical exercises were extracted on the government websites. The results showed that Linshui (48) and Si County (49) organized emergency response drills for COVID-19 on January 15 and April 26, 2022, respectively. The emergency drills in both counties were conducted before the outbreak. And the time interval between the drill and the outbreak was relatively short. To some extent, such exercises were effective rehearsals in response to the large-scale epidemic in both regions later on. Let's take a look at Jiutai again, its epidemic prevention and control emergency drill was held on October 25, 2021 (50). Its drill had the longest interval between the outbreak.

We can see from the results in Table 8 that Si County has the highest score in the learning ability, while Jiutai District has lower scores in emergency drills and specialized learning. This results in a low overall score for learning ability in Jiutai District.

**Table 8:** Learning ability assessment of three counties.

**Indicators**		**Si County**	**Linshui County**	**Jiutai District**
Learning ability	Crisis communication	25	25	25
	Emergency training	15	0	0
	Emergency drill	25	25	15
	Specialized learning	25	25	0
Aggregate score		90	75	40

### Innovation ability of county-level government

The release dates of emergency plans of three counties were crawled. On December 21, 2021, the 58th executive meeting of the county-level government approved the “Overall Emergency Plan for Sudden Incidents in Si County” (51). On April 15, 2022, the official website of Linshui County released the “Linshui County Emergency Response Plan (Trial)” (52). Jiutai was the latest to issue the “Overall Emergency Plan for Emergencies in Jiutai District, Changchun City” until December 2, 2022 (53). Si County also updated its Public Health Emergency Response Plan in July 2021 in a timely manner. The public health emergency response plans for Jiutai District and Linshui County were last updated in 2020. Public information shows that Linshui released the “Emergency Plan for Public Health Emergencies in Linshui County (Trial)” on January 7, 2023 (54). The Health Commission of Si County formulated the “Emergency Plan for Public Health Emergencies” on December 14, 2023 (55). In addition, the special emergency plan for Jiutai was not accessible through public online channels. In terms of time, the overall emergency plans for both Si County and Linshui were issued before the outbreak of a large-scale epidemic. The epidemic in Jiutai district occurred in March 2022, but the overall emergency plan was not issued until November 2022. During this epidemic, Jiutai still arranged relevant crisis management work in accordance with the overall emergency plan issued in September 2014. After the aftermath of the large-scale epidemic, Jiutai did not update its plans in a timely manner, let alone revise special plans specifically for public health emergencies such as the epidemic. Si County and Linshui had already revised their emergency plans before the large-scale epidemic outbreak, and were able to introduce specialized emergency plans to respond to public health events after the crisis, making more adequate preparations for future crises.

At the onset of the outbreak, county-level government in all three regions made timely use of new media platforms to guide public opinion. During the outbreak, the first item in the daily tweets of the WeChat official account “Linshui Release” of the Linshui County Unified Media Center was always a notice about the number of new infections and the level of risk in the region. Jiutai District also announced the number of new infections every day on its WeChat official account “Jiutai Rongmedia.” Si County utilized multiple channels to disseminate information about the outbreak, for example, using the WeChat official account “Si Publishing” to disseminate information about the outbreak. This suggests that county-level government in all three regions have been innovative in their information dissemination channels.

In dealing with the outbreak, the county-level government of the three districts set up special working groups in a timely manner. In order to do a good job of sealing and controlling the district during the people's livelihood protection work, Jiutai District government set up a livelihood protection work task force. Linshui County set up a task force. It divided into sealing area, control area, preventive area to prevent and control COVID-19. Si County has established a set of “five types of responsible persons guarantee contact” community (village) epidemic prevention and control work system. Si County government has perfected the “five types of responsible persons guarantee contact” community prevention and control mechanism of township cadres, grid workers, grassroots medical workers, civilian police, and volunteers. The governments of the three regions have made organizational innovations in the prevention and control of epidemics.

In terms of technological innovation, governments in all three counties used new technologies in the fight against the epidemic. Place codes and health codes were used on a large scale during the outbreak. The county-level government in all three regions used place codes and health codes to automatically register information about people entering and leaving the premises. The use of this technology improved the efficiency of access. In the case of a localized outbreak, the venue code and health code facilitated the relevant departments to carry out accurate traceability and investigation. The development of online teaching and online office technology also greatly reduced the speed of the spread of the epidemic.

As shown in Table 9, the innovation abilities of the three counties are not significantly different. Among them, the score of Jiutai District is slightly lower than the other two counties. The score for institutional innovation in Jiutai District is 0. This is the reason why there is a gap between Jiutai District and the other two counties.

**Table 9:** Innovation ability assessment of three counties.

**Indicators**		**Si County**	**Linshui County**	**Jiutai District**
Innovation ability	Institutional Innovation	25	25	0
	Organizational Innovation	25	25	25
	Technological Innovation	25	25	25
	Information Dissemination Innovation	25	25	25
Aggregate score		100	100	75

## Discussion

In the process of responding to the epidemic, the dynamic capabilities of the three county governments are shown in Table 10. Si County had the highest score, the score in Jiutai District was the lowest. This indicated that the dynamic capability of Jiutai District government was the weakest. From the Table 10, it can be seen that Jiutai District had a medium level of innovation ability. But because of the weak insight ability of Jiutai District government, it pulled down the total score. In the dynamic capacity framework of the government's response to the COVID-19, the four abilities of insight, integration, learning, and innovation did not exist in isolation. They were interrelated and mutually reinforcing, together forming an organic whole. This overall drived the county government to take rapid and precise actions in epidemic prevention and control. The insight capability provided the basis for action for the integration capability. Jiutai District's failure to assess the epidemic situation led to difficulties in integrating subsequent resources. It exacerbated the spread of the epidemic. Learning ability directly supported and promoted innovation ability. Si County and Linshui County promoted the updating of emergency plans through regular emergency drills (innovation capacity). Jiutai District, on the other hand, was also slightly less innovative due to a lack of learning. The innovation capacity then reinforced the effectiveness of the insight capacity on the ground. Linshui County quickly released outbreak information through new media, which improved public risk perception. Innovations in new technologies, such as the promotion of health codes and place codes, also improved the efficiency of prevention and control. The application of health codes and place codes enabled the county-level government to keep track of information about infected people, so that it could make correct decisions. These four capabilities interacted with each other to form a dynamic cycle.

**Table 10:** Evaluation of dynamic abilities in three counties.

	**Weights**	**Si County (raw/weighted)**	**Linshui County (raw/weighted)**	**Jiutai District (raw/weighted)**
Insight ability	30%	75/22.5	80/24	40/12
Integration ability	30%	80/24	60/18	40/12
Learning ability	20%	90/18	75/15	40/8
Innovation ability	20%	100/20	100/20	75/15
Aggregate score	100%	84.5	77	47

From the basic situation of the three districts, Jiutai District had the smallest permanent population and the lowest population density. The outbreak of COVID-19 had the greatest impact on the older adult. In a study analyzing the factors associated with mortality among the older adult in Italian Coronavirus, older adult people over 65 years of age were chosen as a reference (56). The percentage of people over 65 years old in Jiutai District was in the middle of the three regions. In other words, Jiutai District was not the worst in terms of epidemic prevention. However, Jiutai had the worst epidemic prevention effect. This suggests that when a large-scale epidemic occurs, dynamic capabilities of county-level government are very important for effectively responding to crises. Si County and Linshui County have poor conditions for epidemic prevention. However, the governments of the two counties achieved better epidemic prevention and control because they possessed dynamic capabilities. The government of Jiutai District could not make effective epidemic prevention and control due to the lack of dynamic capacity.

After entering the stage of normalized epidemic prevention and control, the central government of China has delegated the leadership of epidemic prevention and control to county-level government. Since ancient times, county-level political power has always been crucial in China. Currently, Chinese national leaders have repeatedly emphasized that county-level governance is the cornerstone of governance. Because county-level government have relatively independent management authority, resource absorption power and decision-making power in the design of the institutional system. County in China's political system is the junction between the state and society, as well as between the government and the people. It can be said that the county is located in the weakest stress area of the hierarchical structure. Some studies have found that social conflicts in China are prone to occur at the most vulnerable points of political stress (57). Therefore, the county has the characteristic of stress vulnerability. After public crisis occurs, the county-level government, as the “front line” in responding to emergencies, directly faces the grassroots masses and plays an important role in emergency management, such as uploading and issuing, early handling, and policy implementation. This further increases the pressure on the county level. If county-level government are unable to effectively address various social problems, including crises, these problems will be transmitted to higher-level government. The crisis that has not been properly handled will spill over to other regions and ultimately require higher-level government intervention to resolve it. Therefore, the governance capacity of county-level government is crucial.

Faced with the large-scale epidemic, the performance of three counties differed significantly. Especially, Jiutai was unable to effectively implement epidemic prevention and control due to lack of dynamic capabilities. The vulnerability of counties in the face of crises can be improved from the perspective of dynamic capabilities, providing valuable experience for county-level government in the face of sudden public crises in the future. First, in terms of insight capacity, county-level government should establish a standing early warning mechanism to shorten response time. The successful experience of Si County shows that rapid release of early warning information and taking action is the key to controlling the spread of an epidemic. County-level government can introduce an intelligent early warning system that integrates data from multiple sectors to monitor potential risks in real time. It can also formulate graded warning standards and clarify response measures under different risk levels.

Second, county-level government should enhance their ability to integrate resources. On the one hand, it is recommended to increase financial investment in the field of public health, especially emergency supplies stockpiling, medical facilities construction and personnel training. And a special emergency fund should be set up for pre-preparation and post-recovery of emergencies. On the other hand, strengthening cross-sectoral collaboration is the key to improving the efficiency of emergency management. Although all three counties have established cross-departmental working groups, the efficiency of collaboration may vary. Therefore, county-level government can establish an incentive mechanism for cross-departmental collaboration to recognize and reward departments and individuals with outstanding performance. They can also establish a clear division of responsibilities and collaboration process to avoid shirking and inefficiency.

In terms of enhancing learning capacity, strengthening emergency training and drills is an important way to improve the dynamic capacities of the government. Si County has demonstrated a strong learning ability by setting up training courses, and Linshui County has demonstrated a strong learning ability by holding several leadership team meetings. This paper suggests organizing regular internal government emergency training and inviting experts to give lectures. It also carries out multi-hazard emergency drills to simulate emergencies in different scenarios and improve the practical ability. At the same time, a training effect evaluation mechanism should be established to ensure that the training content matches the actual needs. In addition, county-level government should take the initiative to learn from external experience. The government can establish a cross-regional experience exchange mechanism and organize regular learning and exchange activities. It is recommended to create an emergency management knowledge base to collect and organize excellent cases and best practices at home and abroad for the government's internal reference.

Finally, governments should focus on the ability to learn from crises, whether in normal or abnormal circumstances. That is to say, learning from past or other regional crises and constantly innovating to cope with unknown future crises (39). With the rapid development of digital technology, the regular application of digital tools has become an important means of enhancing the efficiency of emergency management. The three counties extensively used digital tools such as health codes and web-based offices during the epidemic, effectively reducing the spread of the epidemic. County-level government should promote intelligent emergency management systems, utilizing big data, artificial intelligence, and other technologies to improve the efficiency of risk monitoring and decision-making. The development of mobile applications can facilitate the public's real-time access to information and feedback on issues. In addition, county-level government should establish a dynamic emergency plan update mechanism. Based on actual circumstances and changes in external environment, they should timely adjust the contents of the plan, and develop special emergency plans with detailed response measures for different disaster types. Before a large-scale disaster breaks out, county-level government can use scenario simulation technology and means such as virtual reality to test the feasibility and effectiveness of their plans.

## Conclusion

This paper explored the performance of county-level government in dealing with large-scale epidemics from the perspective of dynamic capabilities, to find directions to improve their crisis management capabilities and alleviate the stress vulnerability of county-level government. By comparing the responses of three county-level government in the context of large-scale epidemics, good insight, integration, learning, and innovation abilities can promote county-level government to handle crises effectively. Furthermore, county-level government are more vulnerable to epidemic prevention. Therefore, this study believes that county-level government should formulate targeted policies and measures based on their actual situation to improve their dynamic capabilities to respond to public crises. In this study, the three cases were analyzed in depth and detail. However, it cannot be ignored that the conclusions drawn from the study based on only three cases have some limitations in terms of generalizability. Due to the limited number of cases, it may be difficult for the conclusions obtained to be fully applicable to all county-level government. Future research could increase the number of cases. This paper only starts the study from public health events. Emergencies include natural disasters, accidental disasters, public health events, and social security events, and future research can focus on the impact of the dynamic capacity of county-level government in responding to other types of incidents.

## Data Availability

The original contributions presented in the study are included in the article/supplementary material, further inquiries can be directed to the corresponding authors.
